# An Enhanced IDBO-CNN-BiLSTM Model for Sentiment Analysis of Natural Disaster Tweets

**DOI:** 10.3390/biomimetics9090533

**Published:** 2024-09-04

**Authors:** Guangyu Mu, Jiaxue Li, Xiurong Li, Chuanzhi Chen, Xiaoqing Ju, Jiaxiu Dai

**Affiliations:** 1 School of Management Science and Information Engineering, Jilin University of Finance and Economics, Changchun 130117, China; guangyumu@jlufe.edu.cn (G.M.); 6221192039@s.jlufe.edu.cn (J.L.);; 2 Key Laboratory of Financial Technology of Jilin Province, Changchun 130117, China; 3 Faculty of Information Technology, Beijing University of Technology, Beijing 100124, China

**Keywords:** DBO algorithm, deep learning, social media, sentiment analysis, natural disaster tweets, emergency management

## Abstract

The Internet’s development has prompted social media to become an essential channel for disseminating disaster-related information. Increasing the accuracy of emotional polarity recognition in tweets is conducive to the government or rescue organizations understanding the public’s demands and responding appropriately. Existing sentiment analysis models have some limitations of applicability. Therefore, this research proposes an IDBO-CNN-BiLSTM model combining the swarm intelligence optimization algorithm and deep learning methods. First, the Dung Beetle Optimization (DBO) algorithm is improved by adopting the Latin hypercube sampling, integrating the Osprey Optimization Algorithm (OOA), and introducing an adaptive Gaussian–Cauchy mixture mutation disturbance. The improved DBO (IDBO) algorithm is then utilized to optimize the Convolutional Neural Network—Bidirectional Long Short-Term Memory (CNN-BiLSTM) model’s hyperparameters. Finally, the IDBO-CNN-BiLSTM model is constructed to classify the emotional tendencies of tweets associated with the Hurricane Harvey event. The empirical analysis indicates that the proposed model achieves an accuracy of 0.8033, outperforming other single and hybrid models. In contrast with the GWO, WOA, and DBO algorithms, the accuracy is enhanced by 2.89%, 2.82%, and 2.72%, respectively. This study proves that the IDBO-CNN-BiLSTM model can be applied to assist emergency decision-making in natural disasters.

## 1. Introduction

Natural disasters are phenomena triggered by the forces of nature, such as sandstorms, hurricanes, or forest fires [1,2]. The constant change of climate makes calamities more frequent [3,4]. Because of the unpredictability, suddenness, and destructiveness, natural disasters have caused significant damage to infrastructure, economy, and society [5,6]. During calamities, Twitter, with its powerful real-time interactivity, makes it convenient for people in affected areas to communicate with the outside world and seek assistance [7,8]. Nevertheless, the public simultaneously has fears, worries, and even resistance, leading to many negative online public opinions [9]. Social stability may be at risk if the government fails to steer and manage these viewpoints effectively [10]. Sentiment analysis of tweets helps decision-makers and researchers infer the possible polarity changes to some extent [11]. Then, some targeted disaster-related information and the progress of emergency management can be released in time [12,13]. This is conducive to guiding public opinions in a positive and benign direction. The disaster prevention and mitigation work will also proceed smoothly. Therefore, this research has crucial practical significance.

Sentiment analysis of tweets identifies whether the polarities are positive or negative [14,15], viewed as a binary classification issue [16]. Relevant research methods are categorized into three types: sentiment dictionary, machine learning, and deep learning. The lexicon-based approach utilizes words annotated with emotional scores to match the content to be analyzed [17]. The final polarity is obtained by accumulating the scores for each word [18]. Positive and negative numbers usually represent positive and negative sentiments, respectively. Researchers commonly use the NRC and VADER lexicons. The NRC lexicon lists the associations between several English words with eight basic emotions and two polarities [19,20]. VADER is another rule-based dictionary [21,22,23]. The sentiment lexicon-based approach is simple to understand and accurately reflects the textual structural features [24]. Nevertheless, identical sentiment words may express different meanings in diverse contexts or domains. Some web neologisms and special terms must also be continuously supplemented to meet the demands [25]. Consequently, this lexicon-based method still has issues with accuracy and applicability owing to limitations in size and coverage [26]. Supervised learning-based approaches [27,28] are more prevalent in sentiment analysis research, including machine and deep learning techniques.

The machine learning-based method trains models to learn features from extensive textual data with sentiment labels [29,30]. The trained models are then used to classify and predict polarity for new test text. Naive Bayes and Support Vector Machine (SVM) are representative machine learning approaches. Naive Bayes is based on probabilistic statistics, which assumes that each feature is independent [31]. Predictions are obtained by learning the conditional probability relationship between textual features and sentiment polarities [32,33]. The advantage of Naive Bayes is that it is computationally simple and performs well on small-scale data. However, the results are not satisfactory when the feature attributes are correlated with each other. SVM is a classification technique that operates on the principle of minimizing the structural risk. It separates diverse categories of textual data by finding a maximally spaced hyperplane [34,35]. SVM is beneficial for handling high-dimensional feature spaces and nonlinear issues [36,37], but choosing the kernel function and regularization parameters is crucial. Despite many advantages, the machine learning-based method usually requires manual feature selection and does not fully utilize the semantic information of the context [38,39].

The deep learning-based method has become mainstream due to complicated textual features’ automatic learning capability [40]. Relevant models go through a process from single to hybrid. Convolutional Neural Network (CNN) and Recurrent Neural Network (RNN) are the most essential approaches. CNN ignores sequential information while focusing on textual local features [41]. RNN can model sequential problems but suffers from the limitation of long-term dependency [42]. As a variation, the Long Short-Term Memory (LSTM) solves the gradient vanishing and explosion issues [43]. The Bidirectional Long Short-Term Memory (BiLSTM) comprises forward and backward LSTM that can recognize preceding or following words to access more contextual information [44]. Considering the advantages of combining various approaches, some hybrid models are proposed, such as CNN-LSTM [45] or CNN-BiLSTM [46]. Experiments demonstrate that hybrid models outperform single models on sentiment polarity classification [47]. Nevertheless, the performance of deep learning models rests on the hyperparameter settings, including the learning rate or the number of neurons [48]. There is no uniform standard. Manual optimization is time-consuming and requires professional knowledge [49].

Swarm intelligence optimization algorithms solve complicated optimization issues by simulating biological behavior in nature [50,51]. To further improve the accuracy of textual sentiment polarity classification, researchers have applied the Grey Wolf Optimization (GWO) algorithm [52,53,54] and Whale Optimization Algorithm (WOA) [55,56,57] to optimize the hyperparameters of deep learning models. The “No Free Lunch” theorem posits that no single algorithm can universally excel at solving every optimization issue [58,59]. That is to say, the algorithm performs well in the current task, but other situations may differ. The Dung Beetle Optimizer (DBO) is a novel algorithm proposed in 2022 [60]. It is characterized by high accuracy and fast convergence [61,62]. Compared with previous methods, the DBO algorithm produces superior results [63,64]. However, obtaining the ideal optimal solution is still challenging. Specifically, the global prospecting and local searching capabilities are imbalanced. There are problems with weak global exploration ability and falling into local optimal solutions easily [65,66]. Therefore, it is necessary to seek improvement strategies for the DBO algorithm.

Here, we can present two motivations for this study:

(1) Hybrid deep learning models are more suitable for tweets’ sentiment polarity classification than single models.
(2) Improved swarm intelligence algorithms can optimize the hybrid deep learning models’ hyperparameters to increase classification accuracy further.

This research’s principal contributions are as below:

- We utilize three strategies to improve the DBO algorithm. First, we adopt the Latin hypercube sampling to update the population initialization process. Second, we integrate the OOA’s global prospecting strategy in the ball-rolling dung beetles’ position update equation. Third, we introduce an adaptive Gaussian–Cauchy mixture mutation disturbance for optimal individuals.
- We construct a CNN-BiLSTM model based on local feature extraction and contextual information understanding abilities. We then use the improved DBO algorithm to obtain the CNN-BiLSTM model’s optimal hyperparameters. These hyperparameters include the 1D convolutional layer’s filter number, the convolutional kernel sizes, and the unit number in BiLSTM’s each LSTM layer.
- We conduct extensive comparative experiments with other single and hybrid deep learning models on natural disaster tweets. The empirical analysis proves the IDBO-CNN-BiLSTM model’s superiority in sentiment polarity classification of natural disaster tweets.

## 2. Literature Review

### 2.1. Natural Disasters

Natural disasters are usually divided into three stages: precursor, occurrence, and recovery. According to the characteristics, the focus of scholars is diverse, corresponding to monitoring and early warning, emergency response, and recovery and reconstruction.

Early monitoring and warning aims at adopting technological means to predict impending natural disasters and notify potential affected areas [67]. Early warning systems are vital in mitigating risks by guiding people to respond appropriately and timely [68]. The rapid development of technology has facilitated the emergence of various early warning systems, such as Earthquake Early Warning (EEW) [69], Drought Early Warning (DEW) [70], Landslide Early Warning (LEW) [71,72], and Flood Early Warning (FEW) [73]. Nevertheless, these systems are only sometimes completely applicable due to the disasters’ complexity. Researchers consider integrating monitoring data and social media to improve understanding of calamity events [74]. Some advanced models have also been constructed to forecast disasters accurately [75].

As a pivotal indicator of emergency response [76], resource allocation is a continuous multi-cycle process that minimizes damage and saves lives by transporting essentials such as food, water, medicine, and tents to disaster areas [77]. From various practices worldwide, many casualties result from material delays and shortages [78]. When natural disasters occur, it is challenging to meet the enormous demands in the affected areas, relying only on the unilateral reserve of the government or rescue organizations [79,80]. By building a game model, each participant will seek a balanced condition of maximizing interests to fully invoke the relief supplies [81,82]. Furthermore, some information technology or neural network algorithms have been utilized in the supply chain to establish a complete system and improve efficiency [83,84].

Post-disaster recovery and reconstruction are complex, dynamic, and multifaceted [85]. The effectiveness and speed depend on the socio-economic characteristics, adaptive capacity, and the response of policymakers [86,87]. During this time, the government needs reliable information to understand the damage extent and formulate recovery strategies [88]. As a powerful tool, remote sensing observation can be combined with machine learning methods to save time and labor [89]. However, the vast impact caused by natural disasters manifests not only in terms of economy and infrastructure but also on the public’s mental health issues [90]. Along with the psychological trauma of experiencing a disaster, people in the affected areas will lose their sense of identity, belonging, and happiness to a certain extent. Even maintaining the basic aspects of daily life is challenging [91]. The support provided by society and the community can help disaster-affected people alleviate mental stress, reduce anxiety, enhance their sense of security, and eventually adapt to the changes in post-disaster life [92,93].

Overall, the work on natural disasters emphasizes the focus of emergency management at diverse stages. These studies prompt us to explore how we can propose specific measures for the government and related departments based on calamities’ evolution, considering the public’s changing sentiments and demands.

### 2.2. Social Media Analysis of Natural Disasters

Compared to expensive and time-consuming traditional survey methods, social media provides convenient ways to obtain public opinions and instrumental disaster information [94]. Social media analysis of natural disasters can be broadly categorized into the following three areas: rumor detection, topic modeling, and sentiment analysis.

Social media has indicated significant advantages in disseminating urgent information [95]. Because of the difficulty of supervision, content on platforms is ambiguous and complex to recognize [96]. In natural disaster events, people are more likely to believe and share unconfirmed information due to intense anxiety and emotional vulnerability [97]. In addition, misinformation spreads faster than actual news, resulting in a specific time lag in dispelling rumors [98]. The prevalence of rumors becomes a critical limiting factor for managers in decision-making. Social stability will be affected, potentially causing delays in implementing disaster response measures. There are currently two main methods for detecting misinformation on social media platforms. One relies on expert manual fact-checking, which is an effective means to combat rumors. Nevertheless, the time cost and human resources required must be considered [99]. The other uses advanced techniques to extract user or textual features [100,101,102], enabling real-time automatic rumor detection.

Investigating the topics of interest on social media can assist managers in understanding the public’s demands and deploying response resources [103]. It is a challenge to mine substantial data for valuable information. Topic modeling helps researchers identify significant themes from textual data [104]. One of the most frequently utilized means is Latent Dirichlet Allocation (LDA), proposed in 2003. LDA is an unsupervised machine learning method for text mining that gives the topic of each document as a probability distribution [105,106]. The performance of LDA in topic modeling of natural disaster data has been proven [107,108]. However, conventional models are limited to short texts, and some words are frequently shared between topics. Accordingly, improved approaches based on domain knowledge have been proposed in recent studies to enhance the accuracy of topic recognition further [109,110]. Moreover, the novel Biterm Topic Model (BTM) is also becoming popular because of its excellent applicability [111].

Sentiment in natural disaster data is equally valuable. Temporal and spatial sentiment analyses help deepen the exhaustive understanding of social responses and provide some essential information for emergency management. By calculating sentiment scores for tweets, quantitative data can reflect emotional evolutionary trends and distribution [112]. From a temporal perspective, public sentiment is constantly in flux during diverse phases of a natural disaster [113]. As the catastrophe subsides and relief efforts are in full swing, sentiment will gradually change from negative to positive [114]. From a spatial perspective, some tweets have geolocation attributes that indicate the user’s location [115,116]. Based on the latitude and longitude, counting the average sentiment value in each region can be studied for visualization and correlation analysis. Especially in some specific domains, social media is more suitable for obtaining sentiment analysis data than Geographic Information System (GIS) techniques [117].

Analyzing social media data during natural disasters is of great research value and practical significance. Nevertheless, few scholars have focused on the sentiment polarity categorization of disaster tweets, and adopting advanced models is even less common. We effectively enhance the performance of hybrid deep learning models by utilizing the improved swarm intelligence algorithm to optimize hyperparameters.

## 3. Method

**Figure 1:**
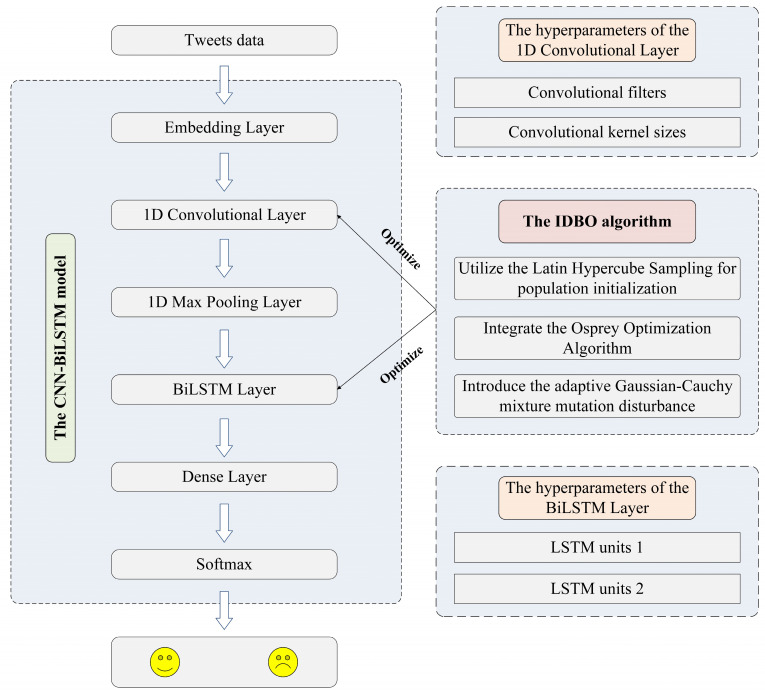
The architecture of the IDBO-CNN-BiLSTM model.

This study constructs an enhanced IDBO-CNN-BiLSTM model for recognizing sentiment polarity in natural disaster tweets. First, the DBO algorithm is improved by adopting three strategies. Then, the IDBO algorithm is utilized to optimize the hybrid CNN-BiLSTM model’s hyperparameters. Finally, the IDBO-CNN-BiLSTM model classifies the sentiment polarity as positive or negative. The proposed model’s architecture is displayed in Figure 1.

### 3.1. The DBO Algorithm

The DBO algorithm simulates the ball-rolling, dancing, breeding, foraging, and stealing behaviors of dung beetles. The algorithm’s population is divided into four parts: ball-rolling dung beetles, brood balls, small dung beetles, and stealing dung beetles. The detailed description is as below.

#### 3.1.1. The Ball-Rolling Dung Beetles

Without obstacles, dung beetles utilize the sun to locate and keep the dung ball rolling in a straight line. The algorithm assumes that the light strength affects the dung beetles’ route. At this point, the location update of the ball-rolling dung beetles is expressed as Equation (1). The parameters are described as shown in Table 1.

(1) $$x_{i}\left(t+1\right)=x_{i}\left(t\right)+\alpha \times k\times x_{i}\left(t-1\right)+b\times ∆x$$

(2) $$∆x=\left|x_{i}\left(t\right)-X^{W}\right|$$

α is determined through a probabilistic approach to emulate the intricate conditions in the natural environment. A greater value of ∆x signifies a less intense light source.

When dung beetles encounter obstacles preventing them from rolling forward, they must dance to reposition. A tangent function simulates this behavior. The location update at this time is calculated by Equation (3).

(3) $$x_{i}\left(t+1\right)=x_{i}\left(t\right)+\tan\left(\theta \right)\left|x_{i}\left(t\right)-x_{i}\left(t-1\right)\right|$$

θ denotes the deflection angle belonging to [0, π]. The location will change if θ equals 0, π/2, or π.

#### 3.1.2. The Brood Balls

Choosing spawning sites is crucial. Dung balls are concealed after being rolled to a safe place. A boundary selection strategy is adopted to model the spawning area of female dung beetles. This region is restricted by Equations (4) and (5).

(4) $$Lb^{*}=max\left(X^{*}\times \left(1-R\right),Lb\right)$$

(5) $$Ub^{*}=min\left(X^{*}\times \left(1+R\right),Ub\right)$$

(6) $$R=1-t/T_{max}$$

After defining the zone, the female dung beetles select the brood balls to spawn. The DBO algorithm assumes that each female dung beetle only reproduces once in each iteration. In addition, the boundary range is dynamically changing, primarily dictated by the R-value. Therefore, the brood balls’ locations are also changeable during iteration. The position update can be calculated by Equation (7). The parameters are described as shown in Table 2.

(7) $$B_{i}\left(t+1\right)=X^{*}+b_{1}\times \left(B_{i}\left(t\right)-Lb^{*}\right)+b_{2}\times \left(B_{i}\left(t\right)-Ub^{*}\right)$$

#### 3.1.3. The Small Dung Beetles

Small dung beetles will drill out of the ground to search for food. The DBO algorithm establishes an optimal foraging area. The region boundaries are restricted by Equations (8) and (9). The small dung beetles’ location update is indicated by Equation (10). The parameters are described as shown in Table 3.

(8) $$Lb^{b}=max\left(X^{b}\times \left(1-R\right),Lb\right)$$

(9) $$Ub^{b}=min\left(X^{b}\times \left(1+R\right),Ub\right)$$

(10) $$x_{i}\left(t+1\right)=x_{i}\left(t\right)+C_{1}\times \left(x_{i}\left(t\right)-Lb^{b}\right)+C_{2}\times \left(x_{i}\left(t\right)-Ub^{b}\right)$$

#### 3.1.4. The Stealing Dung Beetles

Swiping dung balls from other dung beetles is called stealing behavior. The DBO algorithm assumes that the vicinity of $X^{b}$ is the optimal location to scramble for food. The stealing dung beetles’ position update can be calculated by Equation (11). The parameters are described as shown in Table 4.

(11) $$x_{i}\left(t+1\right)=X^{b}+S\times g\times \left(\left|x_{i}\left(t\right)-X^{*}\right|+\left|x_{i}\left(t\right)-X^{b}\right|\right)$$

### 3.2. The Proposed IDBO Algorithm

The DBO algorithm’s advantages are rapid convergence and excellent optimization accuracy. Nevertheless, the global prospecting and local searching capabilities are imbalanced. That is to say, the DBO algorithm suffers from weak global exploration ability and easily falls into local optimization. Consequently, we adopt three improvement strategies to solve the above issues.

#### 3.2.1. Utilize the Latin Hypercube Sampling for Population Initialization

Swarm intelligence optimization algorithms’ convergence speed and accuracy are usually closely related to the initial population’s quality and structure [118]. The random initialization in the traditional DBO algorithm leads to an uneven sample distribution. If the initial population’s quality and diversity cannot be ensured, the algorithm’s searching effectiveness will be significantly affected. Latin Hypercube Sampling (LHS) [119] realizes non-overlapping sampling based on the principle of stratified sampling, which can make the samples evenly distributed in the search space. The updated steps for initializing the population are below:

(1) Determine the number of hyperparameters D representing the optimization problem’s dimension.
(2) Set the range $\left[Lb,\;Ub\right]$ for each hyperparameter, where Lb is the lower boundary, and Ub is the upper boundary.
(3) The range $\left[Lb,\;Ub\right]$ of each hyperparameter is divided into N equal subintervals. N is the population size of the DBO algorithm.
(4) Create a matrix of size N×D. Each column randomly orders the numbers 1,2,⋯,N. Then, a sample is randomly generated in the corresponding subinterval based on the rows’ number. The final resultant forms the initial population.

In LHS, the sample values are usually in the range of $\left[0,\;1\right]$. However, they must be converted to the range set by the corresponding hyperparameters in the optimization problem. The ith sample value of the jth hyperparameter is denoted as:

(12) $$X_{ij}=Lb_{j}+{LHS}_{ij}\times \left(Ub_{j}-Lb_{j}\right)$$

**Figure 2:**
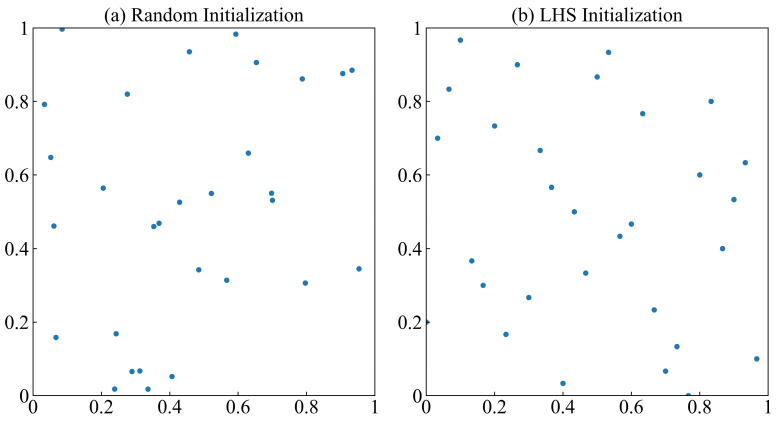
Comparison of two initialization methods.

Assuming a sample size of 30 and a search range of $\left[0,\;1\right]$, the sample distribution in two dimensions is shown in Figure 2. The abscissa and ordinate represent the search scope. The LHS samples are more uniform and have a more extensive coverage than random initialization. Thus, it is proved that using LHS to initialize the population can improve the DBO algorithm’s performance.

#### 3.2.2. Integrate the OOA’s Global Prospecting Strategy

In the traditional DBO algorithm, the ball-rolling dung beetles’ location update strategy depends on the global worst position and has many parameters. Inspired by the OOA [120] proposed in 2023, Equation (1) is improved. The first stage of the OOA is global exploration. Ospreys can detect fish with their powerful vision. After determining the position, the ospreys dive underwater to attack and feed on the fish. The position update in this phase can be expressed by Equation (13). The parameters are described as shown in Table 5.

(13) $$x_{ij}^{P1}=x_{ij}+r_{ij}\times \left({SF}_{ij}-I_{ij}\times x_{ij}\right)$$

During the OOA’s fishing process, the ospreys’ location in the search space changes prominently. If this position update strategy is incorporated into the DBO algorithm, identifying the global optimal region and escaping from the local optimum can be significantly enhanced. Specifically, a more optimal dung ball is randomly selected for rolling during the ball-rolling dung beetles’ position update. The aim is to improve the randomness of the route selection. Equation (14) can calculate the updated location. The parameters are described as shown in Table 6.

(14) $$X_{i}\left(t+1\right)=X_{i}\left(t\right)+rand\left(X^{\prime }-FX_{i}\left(t\right)\right)$$

#### 3.2.3. Introduce an Adaptive Gaussian–Cauchy Mixture Mutation Disturbance

In the traditional DBO algorithm’s later iterations, the dung beetle population will gather and search near the current best location. The algorithm will fail to discover the actual optimal solution if this position is not the global optimum. Performing a mutation perturbation increases the population’s diversity and enlarges the search scope by disturbing the algorithm’s individuals, thus escaping from the local optimum [121]. In other words, the algorithm can enter the solution space’s other regions and continue to explore until it eventually finds the global optimum. Gaussian and Cauchy mutations are two effective disturbance methods. Gaussian mutation is usually based on a normal distribution and explores the solution space by adding small random perturbations in the current solution’s neighborhood [122]. These mutations are symmetrically distributed and form peaks around the mean. The Cauchy variation is based on the Cauchy distribution. This distribution has a sharp peak and a long tail, which can generate more significant perturbations far from the current solution [123]. To combine the advantages, an adaptive Gaussian–Cauchy mixture mutation disturbance is introduced.

The result of the mutation disturbance is randomized. The algorithm’s complexity will increase if all dung beetle individuals are perturbed. Therefore, only the optimal individuals are selected in this study. By comparing the positions before and after the mutation, the better location is chosen for the next iteration. The position after Gaussian–Cauchy mixture mutation disturbance can be expressed by Equation (15). The parameters are described as shown in Table 7.

(15) $$H_{b}\left(t\right)=X^{b}\left(t\right)*\left(1+\mu_{1}*Gauss\left(\sigma \right)+\mu_{2}*Cauchy\left(\sigma \right)\right)$$

(16) $$\mu_{1}=t/T_{max}$$

(17) $$\mu_{2}=1-t/T_{max}$$

Adjusting the weights of Gaussian and Cauchy mutation operators adaptively according to the iterations makes the mixture disturbance more flexible at the algorithm’s diverse stages. Due to the relatively decentralized population distribution, the individuals are mainly perturbed with a more considerable variance by the Cauchy distribution function at the iterations’ beginning. The resulting individuals fully utilize the current location information and increase the random disturbance. As the iteration continues, most individual positions will not change much. At this time, more minor perturbations are applied to the individuals through the Gaussian distribution function. In conclusion, the adaptive Gaussian–Cauchy mixture mutation disturbance can enhance the DBO algorithm’s convergence velocity and even up the local exploitation and global exploration ability.

#### 3.2.4. The IDBO Algorithm’s Time Complexity

Time complexity is an essential metric to measure the algorithm’s efficiency. It describes the performance when the input data may result in the longest running time. A commonly used calculation method is the Big O notation [124]. Define the maximum iterations T, the population size N, and the issue dimension D. The traditional DBO algorithm’s complexity can be expressed as $O\left(N\times D\times T\right)$. The IDBO algorithm is optimized and extended within the original framework and does not change the basic execution order or introduce new loops. Accordingly, the time complexity of the IDBO algorithm remains $O\left(N\times D\times T\right)$. Although the operating efficiency may be affected, the growth rate of the algorithm’s execution time will not vary with an increase in input size.

#### 3.2.5. The Steps of the IDBO Algorithm

The IDBO algorithm’s steps are as below:

**Step 1**: Define the objective function and set the IDBO algorithm’s hyperparameters.

**Step 2**: Initialize the population according to the Latin hypercube sampling. Calculate the fitness values of individuals.

**Step 3**: Set a random number $\delta =rand\left(1\right)$ if the current individual is a ball-rolling dung beetle. When δ<0.9, Equation (14) is used to update the position, incorporating the Osprey Optimization Algorithm; otherwise Equation (3) is utilized. If the current individual is a brood ball, a small dung beetle, or a stealing dung beetle, the location is renewed by Equations (7), (10) and (11), respectively. Boundary detection is performed after each position update.

**Step 4**: Update the current optimal solution and fitness value.

**Step 5**: The current optimum is perturbed by adopting an adaptive Gaussian–Cauchy mixture mutation disturbance to produce a novel optimal solution.

**Step 6**: Repeat Steps 3 to 5. After reaching the maximum iterations, the global optimal solution and fitness value are output.

### 3.3. The CNN-BiLSTM Model

In sentiment analysis, CNN effectively extracts textual local features. BiLSTM is adept at capturing long-distance dependencies and understanding contextual information. The hybrid model can fully utilize the advantages of the two network structures to improve accuracy and efficiency. The CNN-BiLSTM model consists of an embedding layer, a 1D convolutional layer, a 1D max pooling layer, a BiLSTM layer, and a Dense layer. These structures play different roles. The upper layer’s output is the following layer’s input. The 1D convolutional and max pooling layers are used because they apply to sequential data. The details are described below.

#### 3.3.1. Embedding Layer

Before the CNN-BiLSTM model is trained, a vocabulary is usually constructed using the Tokenizer. Each word is mapped to a unique index. Input data is a sequence of word indexes. As the first layer in which the model receives text, the embedding layer’s pivotal role is converting the indexes into a continuous representation of word vectors. These vectors capture and express the semantic information. As a result, the CNN-BiLSTM model can utilize continuous numerical features instead of original textual data for more efficient information extraction and analysis.

#### 3.3.2. 1D Convolutional Layer

The CNN-BiLSTM model’s core is a convolutional layer consisting of multiple convolutional kernels. Each convolutional kernel corresponds to a feature mapping. Specifically, the convolution kernels slid over the input text. The feature mapping is generated by calculating the dot product between the convolution kernel and the textual local region. This process can be expressed by Equation (18). The parameters are described as shown in Table 8.

(18) $$C=f\left(X*K+b\right)$$

#### 3.3.3. 1D Max Pooling Layer

The pooling layer’s primary function is to decrease the feature mapping’s spatial dimensionality. Then, the number of parameters and computations is also reduced. The commonly used operations are max and average pooling operations. The former chooses the maximum as the output. The latter computes all values’ averages and aims to smooth the feature mapping. This study adopts max pooling to retain the most salient features in the textual data and ignore trivial information. The output feature mapping P can be described as follows:

(19) $$P=max\left(C\right)$$

#### 3.3.4. BiLSTM Layer

**Figure 3:**
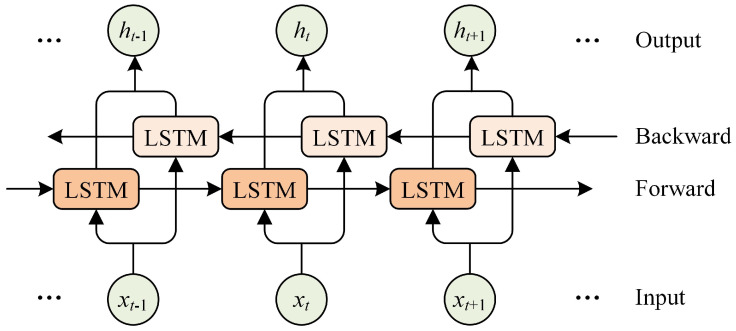
The structure of the BiLSTM network.

The BiLSTM comprises two LSTM layers, one for forward processing and the other for reverse processing. The parameters are updated independently in both directions. The network structure is displayed in Figure 3. When handling the input sequence, the BiLSTM layer combines the extracted local features with contextual information. This particular structure simultaneously considers the words before and after each word in the text, leading to a more comprehensive understanding of the textual meaning. An input gate, a forgetting gate, and an output gate control the info flow of each LSTM unit. The three gates work together in the memory unit. Significant information is learned and memorized, while trivial information is ignored or forgotten. Accordingly, the BiLSTM layer can extract critical features for determining sentiment polarity, such as word order, syntactic structure, and semantic information. The implicit state of the output is expressed as below:

(20) $$h_{t}=\vec{h_{t}}+\overset{\leftarrow }{h_{t}}$$

#### 3.3.5. Dense Layer

The dense layer is located after the CNN-BiLSTM model’s sequence processing section, which integrates the extracted features. The dense layer consists of several neurons. The received input values are multiplied by the corresponding weights. Then, bias is added to obtain a linear combination. An activation function, such as the softmax function, usually follows the dense layer.

## 4. Empirical Analysis

### 4.1. Data Collection and Preprocessing

Twitter contains loads of active users and disaster information, so it can be a data source for sentiment analysis. Meanwhile, Twitter has provided an Application Programming Interface (API) for researchers to access tweets. Hurricane Harvey landed on 25 August 2017, along the southern coast of Texas, USA. This catastrophic event brought extreme rainfall and flooding, causing significant damage and loss of life [125]. Internet users expressed more distinct sentiments than regular events. Therefore, this study selects Hurricane Harvey as the research object. The details of data acquisition are shown below.

**Figure 4:**
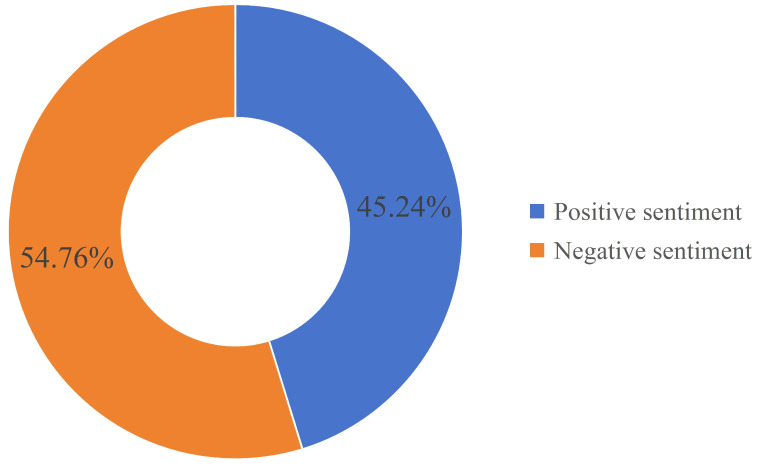
The number of two sentiment labels.

First, this paper utilizes TwitterScraper in Python to obtain data with Hurricane Harvey as the keyword. Second, for a more comprehensive analysis, the data range is extended by one week based on the disaster’s duration. This is because the government often issues disaster warnings in advance, and citizens’ information awareness usually lags. Third, this study collects only English tweets, considering English is a global language. Tweets expressing people’s attitudes towards the relief organizations’ response or their demands are further selected. The aim is to demonstrate that analyzing social media tweets is helpful for more effective disaster management. In the end, a total of 5000 pieces of data are retained. Tweets are manually annotated as positive or negative sentiments. The proportions of the two labels are shown in Figure 4, which are 2262 and 2738, respectively. In this study, positive sentiment is denoted by 0 and negative sentiment by 1. Table 9 cites an instance of the correspondence between tweets and sentiment labels.

Raw data must be converted into a suitable format for analysis or modeling before being fed into the model. The purpose is to eliminate some invalid noise information. The tweet data preprocessing in this study consists of the following tasks:

(1) Remove Twitter handles (@user).
(2) Remove special characters, numbers, and punctuation.
(3) Remove short words with lengths of less than three.
(4) Utilize Tokenizer to segment the text and convert it into a sequence.
(5) Fill the sequence to the same length.

### 4.2. Experimental Details

This experiment is performed on a computer with Python 3.8 and RTX 4090. The training and testing sets are stochastically chosen at a ratio of 7 to 3. L2 regularization is added to the BiLSTM layer to control the model complexity and reduce overfitting. In the IDBO algorithm, the population proportions of ball-rolling dung beetles, brood balls, small dung beetles, and stealing dung beetles are set to 0.2, 0.4, 0.2, and 0.2, respectively. The other hyperparameter settings are shown in Table 10.

### 4.3. Evaluation Metrics

This study adopts four evaluation metrics to compare several models comprehensively, including accuracy, precision, recall, and F1. Higher values represent better classification results. The calculations are expressed by Equations (21)–(24). The standard binary confusion matrix is shown in Table 11.

(21) $$Accuracy=\frac{TP+TN}{TP+FP+FN+TN}$$

(22) $$Precision=\frac{TP}{TP+FP}$$

(23) $$Recall=\frac{TP}{TP+FN}$$

(24) $$F1=\frac{2*Recall*Precision}{Precision+Recall}$$

### 4.4. Experimental Results

#### 4.4.1. The Contrast of Evaluation Metrics

**Figure 5:**
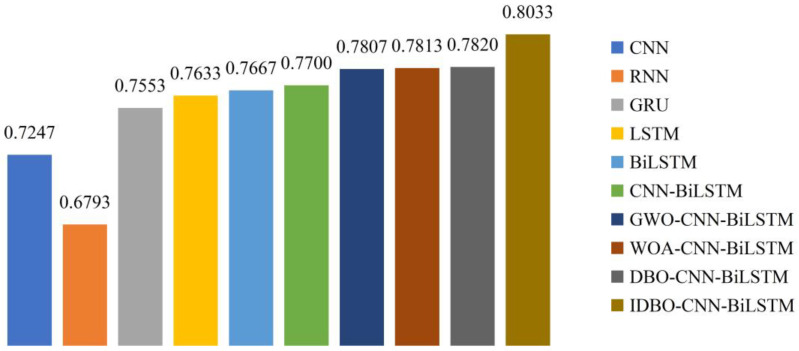
The comparison of accuracy.

Several single and hybrid models are compared to prove the proposed IDBO-CNN-BiLSTM model’s superiority in the sentiment polarity classification. All the experiments are conducted under a consistent operating environment and parameter settings to ensure the results’ reliability. The single models include CNN, RNN, GRU, LSTM, and BiLSTM. The hybrid models are CNN-BiLSTM, GWO-CNN-BiLSTM, WOA-CNN-BiLSTM, and DBO-CNN-BiLSTM. The contrast of evaluation metrics is indicated in Table 12. The comparison of accuracy is displayed in Figure 5.

The results of the evaluation metrics reveal the following findings:

- Among the selected single models, CNN is the only one that can extract textual local features. It achieves an accuracy of 0.7247. The other models are suitable for processing sequential information. Nevertheless, RNN is susceptible to gradient vanishing and explosion. As two variants of RNN, LSTM performs better in capturing long-term dependencies than GRU due to its complex gating mechanism. BiLSTM has a bidirectional LSTM layer that simultaneously considers words before and after each word in the text. The accuracy of BiLSTM reaches 0.7667. Compared to RNN, GRU, and LSTM, BiLSTM improves the accuracy by 12.87%, 1.51%, and 0.45%, respectively.
- The CNN-BiLSTM model, which combines the local feature extraction capability of CNN with the contextual understanding ability of BiLSTM, outperforms both individual methods. The hybrid model achieves an accuracy of 0.7700, increasing by 6.25% and 0.43%, respectively.
- After optimizing the 1D convolutional layer’s filter number, the convolutional kernel sizes, and the unit number in BiLSTM’s each LSTM layer, the performance is better than that of the basic CNN-BiLSTM model. The IDBO algorithm shows the most significant enhancement. The accuracy is 0.8033, improved by 2.89%, 2.82%, and 2.72% compared to GWO, WOA, and DBO algorithms.

#### 4.4.2. The Comparison of Confusion Matrices

**Figure 6:**
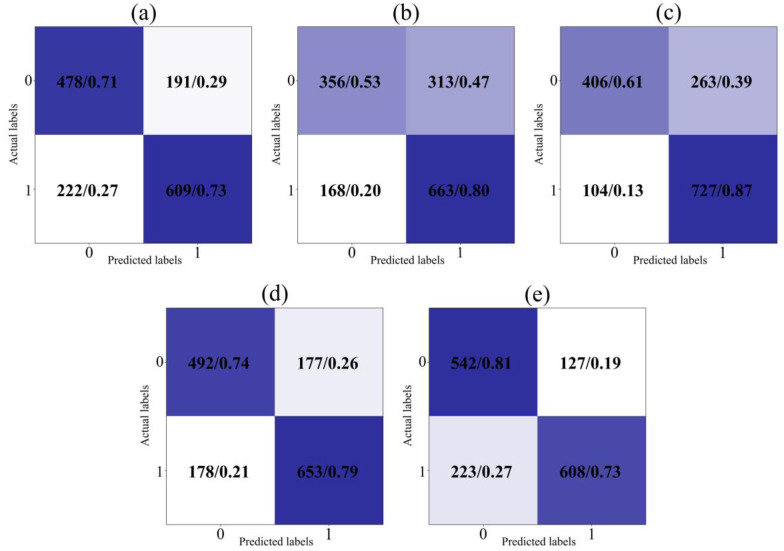
The comparison of confusion matrices for single models: (**a**) CNN; (**b**) RNN; (**c**) GRU; (**d**) LSTM; (**e**) BiLSTM.

**Figure 7:**
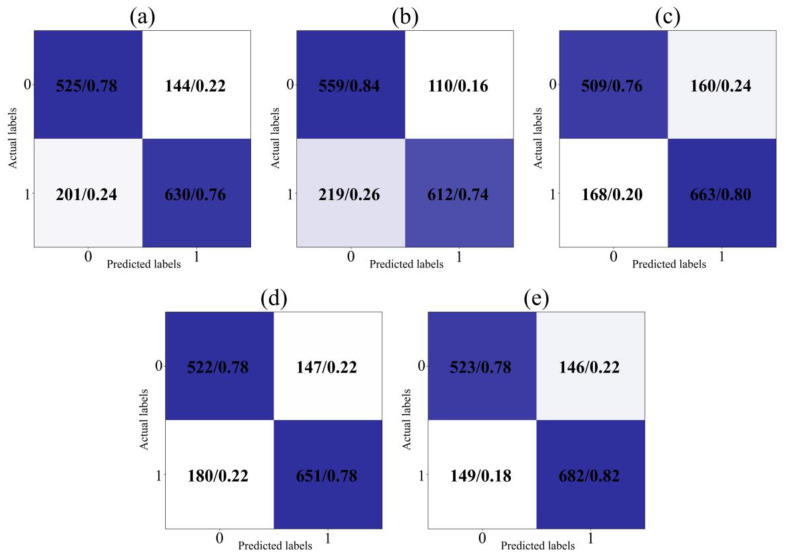
The comparison of confusion matrices for hybrid models: (**a**) CNN-BiLSTM; (**b**) GWO-CNN-BiLSTM; (**c**) WOA-CNN-BiLSTM; (**d**) DBO-CNN-BiLSTM; (**e**) IDBO-CNN-BiLSTM.

The confusion matrix provides an intuitive perspective for comparing the classification of positive (labeled as 0) or negative sentiments (labeled as 1) by diverse models. The contrast results on the test set are shown in Figure 6 and Figure 7. Raw counts indicate the match between the predicted and actual labels. The normalized probabilities are obtained by dividing each cell’s raw counts by the sum of that row or column. Normalized probability makes comparisons between categories fairer because it eliminates the effect of sample size. A darker color means a higher probability.

The IDBO-CNN-BiLSTM model’s accuracy for categorizing positive and negative sentiments is 78% and 82%, respectively. More negative than positive sentiments are expressed in obtained natural disaster tweets. The IDBO-CNN-BiLSTM model, compared with other models, not only maintains a stable accuracy for negative sentiment classification but effectively enhances the categorization performance for positive sentiment. The experimental results demonstrate the proposed model’s superiority in analyzing natural disaster tweets.

#### 4.4.3. The Performance Comparison of Four Optimization Algorithms

The accuracy is noteworthy when utilizing swarm intelligence algorithms to solve optimization problems. Furthermore, time costs also need to be considered. Table 13 shows the optimal hyperparameters and runtime of four models. These algorithms are set with a consistent population and maximum iterations to ensure the experimental results’ comparability.

Table 13 reveals that the IDBO-CNN-BiLSTM model acquires minimal optimal hyperparameter values. As a result, the model will be highly efficient in handling the sentiment classification task. Compared to the WOA and DBO algorithms, the IDBO algorithm takes slightly more time to acquire the optimal hyperparameters. Nevertheless, it is acceptable considering the increase in accuracy.

## 5. Conclusions and Prospect

### 5.1. Conclusions

This study proposes an enhanced IDBO-CNN-BiLSTM model for classifying the sentiment polarity of natural disaster tweets. The hybrid model fully considers the advantages of swarm intelligence optimization algorithms and deep learning methods.

In single models, CNN can extract textual local features. BiLSTM has the most robust ability to process sequence information. This research combines these two network structures to construct a CNN-BiLSTM model. The deep learning models’ performance mainly depends on the hyperparameters. Manual settings increase the difficulty and randomness. Swarm intelligence algorithms are effective in solving complicated optimization issues. The DBO algorithm’s advantages are rapid convergence and excellent optimization accuracy. Accordingly, the DBO algorithm is selected to obtain the CNN-BiLSTM model’s optimal hyperparameters. These hyperparameters include the 1D convolutional layer’s filter number, the convolutional kernel sizes, and the unit number in BiLSTM’s each LSTM layer. Nevertheless, the DBO algorithm suffers from weak global exploration and falls into local optimum easily. There is still room for performance improvement.

Three improvement strategies have been proposed to address the DBO algorithm’s shortcomings. First, Latin hypercube sampling population initialization is employed to avoid samples’ uneven distribution in the search space. Second, the OOA’s global prospecting strategy is fused into the ball-rolling dung beetles’ position update equation to solve the problem of more parameters. Third, an adaptive Gaussian–Cauchy mixture mutation disturbance is introduced to enhance the algorithm’s performance by disturbing the optimal individuals.

Experimental results of sentiment classification on natural disaster tweets show that the accuracy of BiLSTM is improved by 12.87%, 1.51%, and 0.45% compared to RNN, GRU, and LSTM, respectively. The CNN-BiLSTM model outperforms the separate models, with an accuracy enhancement of 6.25% and 0.43%, respectively. The IDBO algorithm has the most remarkable optimization effect among several swarm intelligence algorithms. In contrast with the GWO, WOA, and DBO algorithms, the accuracy is increased by 2.89%, 2.82%, and 2.72%, respectively. Furthermore, the proposed model’s optimal hyperparameters are minimal. Consequently, the IDBO-CNN-BiLSTM model will save more computing resources in sentiment analysis.

In general, this study has momentous practical implications. The proportion of sentiment polarity in actual natural disaster tweets is usually unbalanced. The IDBO-CNN-BiLSTM model’s classification performance is more stable than other algorithms. Comparative experiments prove the proposed model’s superiority in coping with natural disaster tweets.

### 5.2. Suggestion

In the natural disaster tweets obtained, the public’s needs are primarily in the following areas: food, water, housing, transportation, and medical care. If these demands are responded to and met promptly, more positive sentiment will be collected on social media platforms. But overall, the proportion of negative emotion exceeds positive sentiment, indicating that governments or relief organizations still need to enhance their emergency management capabilities. According to the evolution of calamities and changes in people’s requirements, diverse stages should have corresponding focuses. Internet users and social media platforms should also work closely together to minimize the damage caused by natural disasters.

Several potential crisis factors emerge when natural disasters are in the precursor phase. It is a critical period for prevention and preparation. Netizens need to raise their self-protection awareness and prepare emergency supplies immediately after receiving official notifications. Social media platforms should carry out educational activities and push disaster information to users rapidly and accurately. The government and related agencies must establish monitoring systems and formulate detailed contingency plans, including evacuation routes, stockpiling and distribution of relief materials, and training and drills for rescue teams.

Natural disasters in the occurrence phase cause direct damage to human society and the environment. This period is characterized by colossal destructiveness and wide-ranging impact. Internet users need to remain calm and follow official instructions for evacuation or sheltering. They should also avoid spreading unconfirmed information to reduce panic and confusion. Social media platforms can utilize technical means to monitor and manage inaccurate news’ spread, such as keyword filtering or user reporting systems. The government and humanitarian organizations must immediately activate the emergency response plans and concentrate rescue forces to assist the affected areas.

When natural disasters are in recovery process, emergency management efforts include disaster assessment, infrastructure reconstruction, and psychological rehabilitation. Netizens can participate in community work or organize online fundraising and material donation activities. Social media platforms should provide data support for assessing the persistent impact of calamities. The government and relevant departments must prioritize restoring basic facilities and public services. Psychological counseling for the affected population is also necessary. Post-disaster recovery may last a long time and require collaborative efforts.

### 5.3. Limitation and Future Prospect

This paper still has some limitations. The current contents of social media platforms are no longer restricted to plain text. Internet users tend to utilize images to express their viewpoints. Meanwhile, there are some complicated implicit emotions, such as sarcasm. The text or images are opposite to the actual emotional tendencies. To a certain extent, it affects the recognition results. Future research can consider proposing more advanced classification models or performing multimodal sentiment analysis.

## Figures and Tables

**Table 1:** The description of the parameters in Equations (1) and (2).

Parameters	Description
t	The current iteration number
$x_{i}\left(t\right)$	The ith dung beetle’s position information at the tth iteration
α	A natural coefficient assigned as −1 (deviation) or 1 (no deviation)
k	A constant value representing the deflection coefficient in the interval (0, 0.2]
b	A constant value belonging to (0, 1)
$X^{W}$	The worst global position
∆x	The simulation of light intensity change

**Table 2:** The description of the parameters in Equations (4)–(7).

Parameters	Description
$X^{*}$	The current local optimal position
$Lb^{*}$	The spawning zone’s lower boundary
$Ub^{*}$	The spawning zone’s upper boundary
$T_{max}$	The maximum iterations
Lb	The optimization issue’s lower boundary
Ub	The optimization issue’s upper boundary
$B_{i}\left(t\right)$	The ith brood ball’s location information at the tth iteration
$b_{1}$, $b_{2}$	The stochastic vectors by size 1 × D
D	The optimization problem’s dimension

**Table 3:** The description of the parameters in Equations (8)–(10).

Parameters	Description
$X^{b}$	The global optimal position
$Lb^{b}$	The lower boundary of the optimal foraging zone
$Ub^{b}$	The upper boundary of the optimal foraging zone
$x_{i}\left(t\right)$	The ith small dung beetle’s position information at the tth iteration
$C_{1}$	A stochastic value following the normal distribution
$C_{2}$	A stochastic vector belonging to (0, 1)

**Table 4:** The description of the parameters in Equation (11).

Parameters	Description
$x_{i}\left(t\right)$	The ith thief’s position information at the tth iteration
g	A stochastic vector following the normal distribution by size 1 × D
S	A constant value

**Table 5:** The description of the parameters in Equation (13).

Parameters	Description
$x_{ij}$	The ith osprey’s position information at the jth dimension
$r_{ij}$	A stochastic value within the scope [0, 1]
${SF}_{ij}$	The location information of the fish chosen by the ith osprey at the jth dimension
$I_{ij}$	A stochastic value from {1, 2}

**Table 6:** The description of the parameters in Equation (14).

Parameters	Description
$X_{i}\left(t\right)$	The ith dung beetle’s location information at the tth iteration
rand	A stochastic value in the interval [0, 1]
$X^{\prime }$	The selected better position of the dung ball
F	A stochastic value from {1, 2}

**Table 7:** The description of the parameters in Equation (15).

Parameters	Description
$X^{b}\left(t\right)$	The individual’s optimal position at the tth iteration
$\mu_{1}$, $\mu_{2}$	The weight coefficient of the mutation operator
$Gauss\left(\sigma \right)$	The Gaussian mutation operator
$Cauchy\left(\sigma \right)$	The Cauchy mutation operator

**Table 8:** The description of the parameters in Equation (18).

Parameters	Description
f	The ReLU activation function
X	The input word embedding matrix
K	The convolutional kernel matrix
b	The bias term

**Table 9:** The examples of tweets with sentiment labels.

Tweets	Sentiment Labels
Thank you to the many volunteers & farmers from North Dakota who harvested sweet corn & delivered it to the Food Bank for hurricane victims!	0
I need food and water. This freaking hurricane ruins everything!	1

**Table 10:** The hyperparameter settings of this experiment.

Hyperparameters	Value
Optimizer	Adam
Learning rate	0.0001
L2	0.01
Epochs	20
S	0.5
N	10
D	4
Maximum iteration	10
Lb	[3, 32, 64]
Ub	[8, 128, 256]

**Table 11:** The standard binary confusion matrix.

	Predicted Positive Instance	Predicted Negative Instance
**Actual Positive Instance**	True Positive (TP)	False Negative (FN)
**Actual Negative Instance**	False Positive (FP)	True Negative (TN)

**Table 12:** The contrast of evaluation metrics.

Types	Models	Sentiment Labels	Precision	Recall	F1
Single models	CNN	0	0.6829	0.7145	0.6983
		1	0.7612	0.7329	0.7468
	RNN	0	0.6794	0.5321	0.5968
		1	0.6793	0.7978	0.7338
	GRU	0	0.7961	0.6069	0.6887
		1	0.7343	0.8748	0.7985
	LSTM	0	0.7343	0.7354	0.7349
		1	0.7867	0.7858	0.7863
	BiLSTM	0	0.7085	0.8102	0.7559
		1	0.8272	0.7316	0.7765
Hybrid models	CNN-BiLSTM	0	0.7231	0.7848	0.7527
		1	0.8140	0.7581	0.7850
	GWO-CNN-BiLSTM	0	0.7185	0.8356	0.7726
		1	0.8476	0.7365	0.7882
	WOA-CNN-BiLSTM	0	0.7518	0.7608	0.7563
		1	0.8056	0.7978	0.8017
	DBO-CNN-BiLSTM	0	0.7436	0.7803	0.7615
		1	0.8158	0.7834	0.7993
Proposed model	IDBO-CNN-BiLSTM	0	0.7783	0.7818	0.7800
		1	0.8237	0.8207	0.8222

**Table 13:** The optimal hyperparameters and runtime of four models.

Models	Convolutional Filters	Convolutional Kernel Sizes	LSTM Units 1	LSTM Units 2	Runtime (Seconds)
GWO-CNN-BiLSTM	76	4	197	120	3432.0266
WOA-CNN-BiLSTM	128	4	203	131	1711.1468
DBO-CNN-BiLSTM	82	6	177	128	1778.9641
IDBO-CNN-BiLSTM	32	3	64	87	1936.3141

## Data Availability

The raw data supporting the conclusions of this article will be made available by the authors on request.
